# Tactical expertise assessment in youth football using representative tasks

**DOI:** 10.1186/s40064-016-2955-1

**Published:** 2016-08-09

**Authors:** Jaime Serra-Olivares, Filipe Manuel Clemente, Sixto González-Víllora

**Affiliations:** 1 Departamento de Pedagogía en Educación Física, Facultad de Educación, Universidad Católica de Temuco (Chile), Rudecindo Ortega, 02950 Temuco, Chile; 2 Instituto Politécnico de Viana do Castelo, Escola Superior de Desporto e Lazer, Melgaço, Portugal; 3 Teacher Training Faculty of Cuenca, Universidad de Castilla-la Mancha, Cuenca, Spain

**Keywords:** Tactical knowledge, Assessment, Small-sided and conditioned games, Representative task, Expertise, Football

## Abstract

Specific football drills improve the development of technical/tactical and physical variables in players. Based on this principle, in recent years it has been possible to observe in daily training a growing volume of small-sided and conditioned games. These games are smaller and modified forms of formal games that augment players’ perception of specific tactics. Despite this approach, the assessment of players’ knowledge and tactical execution has not been well documented, due mainly to the difficulty in measuring tactical behavior. For that reason, this study aims to provide a narrative review about the tactical assessment of football training by using representative tasks to measure the tactical expertise of youth football players during small-sided and conditioned games. This study gives an overview of the ecological approach to training and the principles used for representative task design, providing relevant contribution and direction for future research into the assessment of tactical expertise in youth football.

## Background

Invasion games such as basketball, handball and football, among others, carry a high level of uncertainty and time pressure, mainly caused by the cooperation-opposition relationship. Uncertainty is an important element strongly linked to time. A good information process and quick decision-making are key elements to reduce uncertainty (Gréhaigne et al. 1997). For that reason, the objective for each team is to reduce uncertainty for itself and, at the same time, to increase uncertainty for the opposite team. The reality of evolving game play offers a very large variety of concrete game situations in connection with the notion of opposition relationship. This is a constantly changing environment that includes a set of problems that need to be solved (Davids et al. 2006). It presents a constant challenge to the athletes, developing their ability to adjust to each situation, developing technical and tactical skills appropriate to each case, and acquiring so-called tactical expertise (Araújo et al. 2006). In this way the perceptual and cognitive components related to tactics have gained great importance in the teaching of sport and the assessment of learning (Dicks et al. 2008).

Despite the vast research in previous years into teaching processes based on ecological perspectives (Araújo and Davids 2009; Correia et al. 2012), a much smaller amount of investigation has been dedicated to representative tasks used to assess the tactical expertise of youth football players. Representative tasks could be defined as carefully structured and managed teaching–learning drills to maintain relationships between key sources of information and action for learners and performers during practice (Brunswik 1956; Chow et al. 2006). Nevertheless, using representative tasks for assessment is suggested as an appropriate methodology which correctly evaluates tactical expertise in players who have participated in a teaching program based on small-sided and conditioned games (Davids et al. 2006). For this reason, the aim of this narrative review is to analyze the investigations that have been conducted on this topic and to identify the main pedagogical and didactical principles to best design representative tasks to specifically measure the tactical behavior of football players.

We searched the electronic databases of PubMed, SPORTDiscus and Google Scholar for potential papers. The following keywords were used in multiple combinations: “small-sided games”, “small-sided and conditioned games”, “drill based activities”, “task design in soccer”, “representative tasks”, “skill-based training”, “skill-based assessment” and “ecological assessment in soccer”. Since the focus of this review is on game design and the lack of studies on this sport, we did not apply any limits to the search period.

## Tactical expertise and performance in sport

The development of sports tactics expertise is a performance requirement in all sports. This quality not only involves the ability to determine whether a strategy used during sports activity is the most appropriate, but also whether this strategy can be properly executed within the limitations of the required movement (Stratton et al. 2004). A player’s ability to anticipate the opposing team’s responses can bee explained by their (perceptual-cognitive) visual and decision-making skills.

Regarding visual abilities, the most skilled players have been proven to excel over less skilled players in certain conditions (Mann et al. 2007), although there have also been some points of disagreement on this topic (Helsen and Starkes 1999; Ward and Williams 2003). Although it is true that the quality of sensory information that is gathered by the eye is a decisive quality for anticipation in sport, it seems that the way this information is perceived and treated by the player has a deep and lasting influence on the actions used to achieve success (Ericsson 1996; Williams et al. 1999).

Those athletes who have a higher expertise level have shown a greater ability in using visual information that has been obtained from their opponents, suggesting higher levels of learning in relation to tactics (Ward and Williams 2003). Players’ visual observations are organized into different information areas according to each high-informational tactical moment, and the indexes which enable them to detect, interpret and predict what is going to happen next can be quickly isolated (Starkes and Ericsson 2003).

Likewise, those athletes with higher expertise levels have a greater ability to identify patterns of play, since they possess qualities that allow them to develop more sophisticated knowledge structures through the experience and processes of information retrieval (Starkes and Ericsson 2003). Based on the faster process of information collection that these athletes possess, and taking into account their previous experiences, they experience an easier and more accurate anticipation process than novice players (Ward et al. 2000).

Those higher-level tactical expert athletes (Ruiz-Pérez et al. 2006) are defined as individuals who: (a) are experts in a sport, despite not demonstrating a universal expertise in sport; (b) do not demonstrate expertise in the general measures of individual ability; (c) are more sensitive to and more easily able to recognize play patterns in their sport, allowing them to solve problems that arise earlier and more effectively; (d) can detect and locate better relevant information in their sport; (e) better familiarize themselves with and anticipate the actions of their opponents, objects, and situations (f) have refined self-control skills; (g) have a high level of technical achievement; (h) show a range of excellent behaviors in competition; (i) evaluate their own performances automatically.

In fact, athletic performance can be defined as the successful interaction of multiple faculties to make decisions and execute skills in response to the tactical game (Gréhaigne and Godbout 1995). Therefore, tactics have emerged as the key elements of the new methods of sports teaching, based on the current theories of ecological psychology and dynamical systems (Chow et al. 2007), and these tactics are oriented towards the development of expertise in perceptual-cognitive components of performance (Renshaw et al. 2009). For this reason, tactical expertise evaluation gives a different perspective on the decision-making process in sport (González-Víllora et al. 2015a, b).

## Ecological psychology, dynamical systems and tactical expertise

Aspects of ecological psychology and dynamical systems have contributed to the assessment of tactical expertise in sport, ever since the recognition of neurobiological movement coordination (Kelso 2012). This approach positions the athlete as a complex dynamic system made up of several subsystems. These subsystems are continually interacting in order to make consistent decisions and movement patterns during the game (Travassos et al. 2012; Vilar et al. 2014).

From this perspective, the athlete and the game in which they develop their actions conform to a continuous system that interacts mechanically and informationally (Araújo and Davids 2009). Therefore, the result is the sum of the degrees of freedom of movement in the control process and the intentional adjustments that are made to the constraints of the context game for the development of a particular task (Newell 1986). Thus, the tactical expertise depends on the interactions that occur between individual constraints, the tactical game context, and the ability to develop adaptive skills. Therefore, when assessing efficient play behaviors in sport, there is a need to understand the ecology of these interactions.

## Tactical expertise and the perspective of conditioned motor learning

According to ecological psychology and dynamical systems as applied to sport, performance depends on the intentional adaptations that have been made by athletes facing different sports and different individual conditions, the context, and the task (Araújo et al. 2006; Kugler et al. 1982). The interaction between these factors is what determines the emergence of movement patterns in every moment of the game, because these factors reduce the degrees of freedom of the system (athlete), limiting certain results or specific responses, depending on the context (Bernstein 1967; Newell 1986; Vickers 2007).

**Fig. 1:**
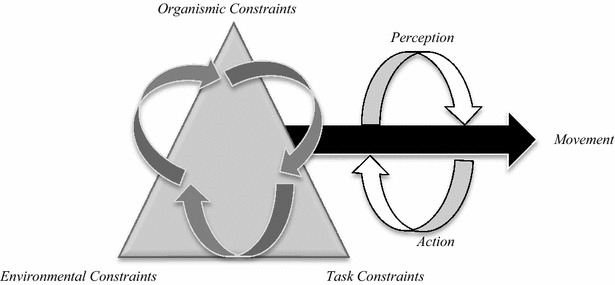
Newell’s constraints model (1986)

As can be seen in Fig. 1, Newell (1986) describes *the individual conditions* as the cognitive, physical, emotional, etc., states of each athlete (including anthropometric characteristics, fitness) delimiting certain movement responses. The constraints of context are referred to as the environment and physical factors such as light or weather conditions. Finally, the constraints of the task refer to the characteristic elements of the situation, actions that are required to be developed, goals, or the number of players, among other things.

Each sports behavior that is developed for a specific purpose results from the athlete’s intentions to meet the task constraints. Thus, flexibility, variability, and behavioral adaptability to the interactions that occur between these factors are said to depend on the tactical and contextual dynamics which develop actions (Passos et al. 2008).

In invasion sports like football, players make decisions and execute skills in two distinct phases; i.e. the attack phase, when the team keeps possession and control of play, trying to create situations that result in the completion of the objective; and the defense phase, when the team is not in possession and tries to regain control, impeding their rival’s and the ball’s progress towards their goal, in order to prevent the opponents from achieving their objective (Gréhaigne et al. 2005). Thus, tactical expertise is influenced by variable dynamics and adaptation to changing contexts. It is a complex dynamic, defined by the internal logic of sport and the sports category in question, including the interests of the participants and the rules of action of the sport, i.e. the tactical problems of game (Bayer 1992).

## Tactical expertise and the contextual dynamics of invasion games: tactical problems

Tactical problems that emerges in the game may constraint the dynamics of the teams (González-Víllora et al. 2011a, b; Vilar et al. 2014). More particularly, tactical problems influence task constraints which have an important effect on the ability of players to achieve objectives and on the collective action of the team (Bayer 1992; Serra-Olivares et al. 2015a).

In team sports such as football, there are until ten attacking and defensive operational principles for tactical behaviors that oppose each other (i.e., see Teoldo et al. 2009). For invasion games teaching these principles are reduced to six attacking and defensive tactical principles (Bayer 1992; Mitchell et al. 2006). In the attacking process, the principles are: (a) keeping possession of the ball; (b) moving forward; and (c) scoring. In the defensive process, the principles are: (a) taking the initiative; (b) preventing the opposition from advancing, and (c) protecting the goal. Based on these principles and their contextual dynamics, new teaching approaches try to develop the tactical perception and action skills of players (Tan et al. 2012). For this reason, it is important to design drills based on the most important factors that lead to the tactical learning of main principles and to try to augment the player’s perception of specific dynamics of the game (Brunswik 1956; Travassos et al. 2011).

It follows that the design of drills should obey detailed pedagogical principles linking the design of tasks to appropriate pedagogical content for team sports teaching such as football (Chow et al. 2007). Recent studies proposed the following pedagogical principles (Griffin and Butler 2005): (a) sampling; (b) representation; (c) exaggeration; and (d) task complexity.

### Sampling

**Fig. 2:**
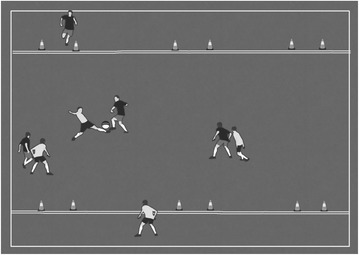
Example of the principle of sampling in a drill. The aim of this drill is to pass the ball between the mini-goals to a teammate. The didactical goal is to teach the player how to move to receive the ball

Sampling advocates that the drills must provide multiple experiences that allow students or players to understand the similarities and specificities of different team sports (Almond 1986; Mitchell et al. 2006). For example, the movement to receive the ball is one of the main characteristics of any invasion team sport, and thus applicable to different sports such as football, basketball or handball (Clemente 2012). Figure 2 presents a sampling example.

Figure 2 represents a task designed to teach football. Nevertheless, when a student or player learns this tactical content, it will allow them to understand the same dynamic in basketball, for example. Therefore, the task constraint used in this game will promote the improvement of perception in order for the player to move between zones to receive the ball and create a line of pass to their teammate who has possession of the ball. Once the player acquires this tactical knowledge, it will be possible to transfer this learning to similar situations in other invasion sports (García-López et al. 2009; Tan et al. 2012).

### Representation

Representation means the modification of the game to keep the main properties of the original format, but at the same time provide a new perspective for the student or player (Almond 1986). In invasion sports, the most common approach is to develop small-sided and conditioned games (Clemente et al. 2014a, b; Davids et al. 2013). The aim of this representation is to provide opportunities for players to develop decision-making processes in specific conditions that are not as recurrent in the original format of the game (Tan et al. 2012).

Representation also keeps the information-movement coupling of the original game’s dynamic, ensuring the ecological integrity of the game (Araújo et al. 2006). Thus, this pedagogical principle remains despite the simplification of the game, avoiding the decomposition that can be typical of analytic approaches to the teaching process (Davids et al. 2006). In short, the representativeness of the task must be based on a close relationship between the dynamic of the modified task and the dynamic of the original format of the game (Tan et al. 2012).

### Exaggeration

**Fig. 3:**
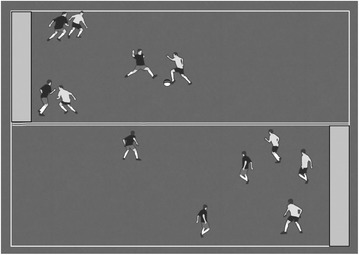
Application of the exaggeration principle. Description: In two isolated zones the ball must circulate from the defensive zone to the attacking zone. The aim is to augment the breadth of the player’s perception of ball circulation. The team scores when a player dribbles across the end zone

The teacher or coach must determine the tactical problem that emerges from the task condition presented in each simplified game (Clemente et al. 2014a, b). These task conditions must guide the players to perform the behaviors determined by the coach. Therefore, exaggeration aims to keep the dynamic of the game and, at the same time, augment the players’ perception of a specific tactical content by using a specific constraint. Figure 3 is an example of the exaggeration principle.

This task condition exaggerates the reality by changing some rules of the game but also keeps the main dynamic of the game. Therefore, this small-sided and conditioned game ensures the dynamic cooperation-opposition process and at the same time creates routines to encourage ball passes for the wings. Thus, players repeat without repeating, based on the variability of the game.

Another important action carried out by the coach is questioning. This process allows the coach to guide players to the answer and can be used to confirm their acquisition of tactical knowledge (Light 2003; Webb and Pearson 2008). Therefore, the feedback provided by questioning complements the didactical processes of design drills.

In summary, representation involves the use of games with the same structure as the competition sport but which have been modified by reducing the structural factors of one or more game elements (the size of the goals, the playing area, the number of players, etc.). An example of a game modified by representation is a 5 a side football. Tactics are similar to the competition game but are adapted to the learners needs. On the other hand, exaggeration involves the modification of key factors of the game. Whereas the essence and the rules remain the same, the purpose is that learners need to face the tactical problems of the sport/category of sport they are learning. An example of a small-sided game modified by exaggeration is a “keeping possession game” in which goals are removed and the purpose is only to achieve passes by using getting free movements (Serra Olivares et al. 2011).

### Task complexity

Task complexity aims to match the drill to the needs and potential of students or players (Lee et al. 2014). Generally, tactical complexity must be progressively increased based on the tactical acquisition of students or players and their increased capacity to be aware of new issues (Hastie et al. 2009).

**Fig. 4:**
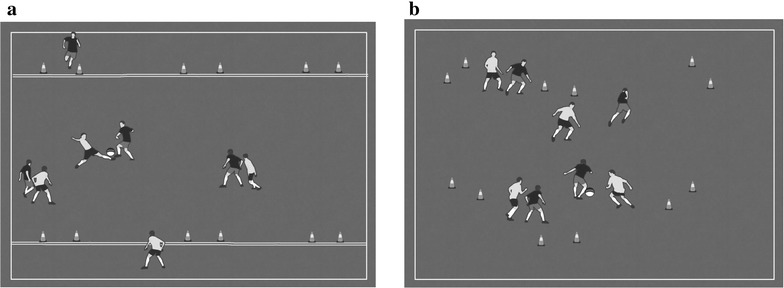
Example of task complexity. **a** The same game of Fig. 3; **b** the aim of this game is to receive the ball between mini-goals, but using movement in any direction. There is no neutral player (floater) and any teammate may receive the ball

One of the main factors that influences complexity is the format of the game. In football, smaller formats (2 vs. 2 or 3 vs. 3) reduces the variability of the game and increases the individual participation of each player (González-Víllora et al. 2011a). Bigger formats increase tactical complexity by increasing the variability of actions and the possibilities of play (Clemente et al. 2014a, b). Moreover, within the same format there may be other task conditions that increase or decrease tactical complexity; thus it can be possible to progressively increase the complexity of the task. Figure 4 shows an example of a pedagogical progression using the tactic of movement to receive the ball.

Task (a) restricts the defenders to the main area of play and there are neutral players that can move freely through their zones of reception. In task (b), multiple points of reception in a unique area increases the complexity for the players and also for the defenders. Thus, task (a) constrains actions and behaviors and task (b) gives more freedom, thus increasing the complexity of the game. In short, within the same format and using the same tactical content, it is possible to simplify or complicate the task by using different task conditions. It is also important to consider that different pedagogical principles may be used at the same time in the development of drills; thus coaches are responsible for using the different features consistently.

## Representative task design, ecological psychology and dynamical systems: Assessing tactical expertise in sport

The practice of task design in sport has changed. The process has been reformed from the utilization of analytical and blocked practices (which are decontextualized) to the use of situations which are representative of sport behaviors. These tasks aim to simulate the performance context in which specific skills are required (Chow et al. 2006). This perspective has its foundation in ecological psychology and dynamical systems theories, and also in the constraints-led approach of motor learning (Araújo et al. 2007; Renshaw et al. 2010). In terms of teaching processes, this approach is based on the idea that with the use of situations that simulate the sport performance context, performers may improve their capabilities for developing flexible behaviors appropriate to the individual, environment, and task constraints (Tan et al. 2012). In the case of representative task design for tactical expertise assessments, the foundation is similar (Davids et al. 2006; Pinder et al. 2011).

Twelve key theoretical principles from ecological psychology and dynamical systems theories can be identified that support the representative task design philosophy for tactical expertise assessment in sport. These principles have been used before to enhance representative task design for teaching processes (Renshaw et al. 2009).

1. If a sport behavior needs to be precisely assessed, the performance situation needs to be effectively simulated. This process must include specific information provided to the player, the response possibilities to the motor problems which are being faced (*affordances*), and the tactical-context dynamics of the game. Thus, it is necessary to understand *the mutuality between the player and his or her context*.
2. *Perceptive elements and movements are coupled.* In this regard, assessment of situations should lead players to perceive all the relevant information regarding suitable responses, as happens in the performance of actions.
3. Motor behavior solutions emerge from the interaction between individual and team constraints. In the cases in which there is tactical equivalence, responses may be similar (stable), although if context dynamics are altered, responses will also be different due to *the capability of the player to self*-*organize and re*-*organize their movements depending on the constraints*. For this reason, it is necessary to design situations representative of real competitive performance when decision-making is being assessed.
4. Performance responds to a multidimensional process of different interacting sport constraints, including tactical-context dynamics. Thus, it must be taken into account that *game behaviors are non*-*linear processes*, and for this reason it is important that they be evaluated in non-linear sport situations (i.e., small-sided games which are tactically representative of the full game).
5. Players act as complex dynamical systems that adapt their behaviors in a flexible manner to the changes imposed by the context. In this respect, *variable contexts of performance are critical for skill development*. Thus, the study of decision-making emergence should faithfully represent the contextualized performance situations.
6. *The player is the main protagonist in his or her assessment*. Thus, the evaluation of tactical expertise must consider individual constraints within the assessment environment. Understanding the intrinsic dynamics as a part of the complex dynamical systems will lead to the design of relevant evaluation contexts which are suitable for the characteristics and needs of the players.
7. The tactical-context of invasion games such as football is altered by the tactical-context problem which is implicit in each game situation. At the same time, *each team is aligned with a different open*-*dynamic system that is the basis of the individual’s tactical behavior emergence*. For this reason, the player’s behavior needs to be understood in the context of the different and changing tactical-context dynamics in which the teams are involved (repetition without repetition situations).
8. Tactical expertise, similar to sport learning, depends on the transition processes of the systems (players) to adapt to the specific provisions of constraints. This factor highlights the need to assess the decision-making skills within situations which are representative of the game tactics, and that involve adaptive flexibility of the player’s behaviors. Thus, it is important to know how *the stability*-*instability of game dynamics influences the quality of the assessment processes*.
9. *The players develop co*-*adaptive movements during the game*. In this sense, the evaluation of tactical expertise while respecting the ecology of the competitive situations, including the different forms of primary constraints (i.e. distances between teams) and secondary constraints (i.e. constraints that emerge from teammates and opponents) is recommended.
10. Sport performance depends on capabilities such as flexibility and adaptability of the systems (players). In this regard, *the creativity of the decision*-*making process during the assessment should be promoted*, highlighting the emergence of functional responses to the exploration and discovery of the technical-tactical possibilities.
11. It is necessary to use tasks that allow individuals to discover problems without providing continuous instructions. This process will *support the emergence of natural and implicit answers* during the assessment of tactical expertise, which gives a stronger representation of the performance situation.
12. The assessment of tactical expertise requires situations to be designed that simulate natural performance contexts in which behaviors are developed within the game. These are *open practice situations* in which players interact with teammates, opponents and the tactical constraints of the game. Using this perspective, the capabilities of the systems (players) to adapt their responses to problems by using flexible decision-making can be analyzed.

Consequently, the representative task design process to assess tactical expertise can be summarized with four premises from the perspective of dynamical ecology (Brunswik 1956; Davids et al. 2006): (a) the player must be provided with both primary and secondary constraints (Renshaw et al. 2009), and there should be no unilateral assessment of behavioral factors (mind, body, and context); (b) the primacy of perception must be recognized, ensuring assessment situations in which perceptual systems are selecting relevant information for the movement; (c) the players must be provided with situations that allow them to adapt their behaviors to the context dynamics (tactical problems), allowing complex interactions between perceptions, intentions and actions; (d) the emergence of adaptive behavior must be facilitated by ensuring that players experience motor problems with ecological links between information and movement. An applied example of this approach is presented in the following section: the evaluation-games design strategy based on the tactical problems of invasion games.

## The tactical-context problem and game design for assessing tactical expertise: An example in youth football

The study of the relationship between individual constraints and task constraints has led to improvements in representative task design for tactical expertise assessment (Araújo et al. 2006; Serra-Olivares et al. 2015b). In this sense, the use of modified games which are representative of the main tactical game and its problems is an ideal strategy for the evaluation of game awareness and the decision-making process (Memmert 2010; Memmert and Roth 2007; Unnithan et al. 2012).

This approach—in which there are similarities between the testing conditions and the constraints of the driving behaviors to be evaluated (Renshaw et al. 2007; Tan et al. 2012)—allows physical education and sports professionals to simulate environments with intra-ecological correlations that are similar to the correlations which occur in the real context of performance (Brunswik 1956; Davids et al. 2006; Vilar et al. 2012). Thus, the decision-making and technical-tactical skills assessment of the players is facilitated, providing relevant information about the learning levels of the athletes (Gutiérrez-Díaz et al. 2011b; Serra-Olivares et al. 2015b).

Representative task design for tactical expertise assessment can be summarized in four phases which are intimately related to the four premises discussed in the previous section of this document: (a) appropriate selection of tactical problems on which the evaluation-game is going to be based and the identification of the specific skill or skills that will be assessed. This requires that players are provided with ecological constraints (primary and secondary) that facilitate a mind–body-context related environment; (b) construction of the test situation based on the selected tactical problem or problems, ensuring a performance-simulated context in which the standard perceptual systems are required; (c) ensuring the representativeness of the tactical problem which is intended to be simulated, allowing the development of information-movement couplings and contextualized perceptual, decision-making and execution skills; (d) ensuring the design of conditions that involve the development of information-movement couplings and the emergence of flexible and functional behaviors. Finally, the quality of the task (the evaluation-game) for assessing the factors—perception, decision making, execution, and other relevant indicators of the skill—will be analyzed.

**Fig. 5:**
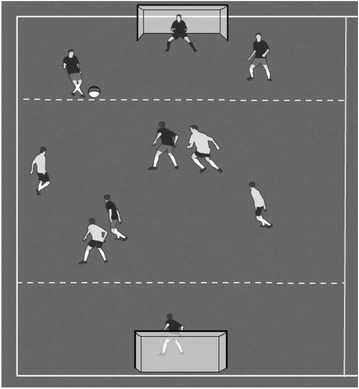
Representative task for the tactical expertise assessment of getting-free, passing, dribbling and shooting skills, in order to penetrate the defense and attack the goal. In the evaluation-game “Shooting without the defense” two teams of four players plus two goalkeepers (4 vs. 4 + 2GK) attack the opponent’s goal and defend their own goal. A goal is achieved when an attacking player dribbles the football into the “scoring zone” (behind the discontinuous line) and scores a goal in the opponent’s goal. The peculiarity of the game is that defenders are forbidden to cross into the “scoring zone” once the attacking player has entered it with the football. Thus, situations of identifying and occupying free spaces are exaggerated for attacking off-ball players. Likewise, passing situations and, most importantly, shooting skills are exaggerated in this context

Figure 5 shows an example of an evaluation-game which represents the dynamics of tactical situations for the development of skills required for advancing t and scoring problems. The purpose of this example is to evaluate the tactical expertise of the players in relation to the skills of getting free, passing, dribbling, and shooting, which are needed during problem solving behaviors within the tactical problems of penetrating the defense and attacking the goal. The principles of dynamical ecology have been used to simulate and to exacerbate the tactical contexts selected for this evaluation in this particular invasion game. Specifically, the number of players, the game size, and some action rules have been adapted according to the proposals of some experts in the field (Mitchell et al. 2006) and the findings of several studies on the topic (González-Víllora et al. 2011b; Serra-Olivares et al. 2015b).

As is shown in Fig. 5, representative task design for tactical expertise assessment contributes to the use of organic constraints during the active scanning of the task by the players (Travassos et al. 2012). Thus, it is possible to evaluate the decision-making processes at the level of the player-context relationship (Passos et al. 2008). Likewise, the situation allows for the generation of perceptual abilities due to the fact that task constraints are simulating the determining factors of the performance skills that are going to be investigated (Davids et al. 2006).

In addition, the dynamic nature of the game facilitates interaction between the perceptions, intentions, and actions of all those involved, because everyone is invested in achieving similar technical and tactical goals. Thus, it is advantageous to design an uncertain environment in which the players must perceive, decide, and act differently in each specific case, depending on the tactical problems of each situation (González-Víllora et al. 2015a, b; Serra-Olivares et al. 2015b). This whole process results in a simulation context that enables information-movement couplings as adapted tactical behaviors to each situation or problem are being developed (Pinder et al. 2011; Unnithan et al. 2012). In short, an ecological environment is achieved in which it is possible to assess the degree of learning by players and teams in the selected decision-making skills.

## Conclusion

Representative tasks to assess the tactical expertise of youth football players may lead to a better understanding of the real status of learning of a player. This survey provided valuable information, principles and guidelines in order to identify the main priorities during assessment. By using a representative task based on the most important criterion of evaluation, it is possible to determine the exact expertise level of players and to then improve the training plan so that it is adequate to the players’ needs. The assessment and training plan form a constant and interactive cycle and, for that reason, an appropriate representative task increases the effectiveness of coaches during training.

## References

[CR1] Almond L, Thorpe RD, Bunker DJ, Almond L (1986). Reflecting on themes: a games classification. Rethinking games teaching.

[CR2] Araújo D, Davids K (2009). Ecological approaches to cognition and action in sport and exercise: ask not only what you do, but where you do it. Int J Sport Psychol.

[CR3] Araújo D, Davids K, Hristovski R (2006). The ecological dynamics of decision making in sport. Psychol Sport Exerc.

[CR4] Araújo D, Davids K, Passos P (2007). Ecological validity, representative design, and correspondence between experimental task constraints and behavioral setting: comment on Rogers, Kadar, and Costall (2005). Ecol Psychol.

[CR5] Bayer C (1992). Invasion games teaching.

[CR6] Bernstein NA (1967). The control and regulation of movements.

[CR7] Brunswik E (1956). Perception and the representative design of psychology experiments.

[CR8] Chow JY, Davids K, Button C, Shuttleworth R, Renshaw I, Araujo D (2006). Nonlinear pedagogy: a constraints-led framework for understanding emergence of game play and movement skills. Nonlinear Dyn Psychol Life Sci.

[CR9] Chow JY, Davids K, Button C, Shuttleworth R, Renshaw I, Araujo D (2007). The role of nonlinear pedagogy in physical education. Rev Educ Res.

[CR10] Clemente FM (2012). Princípios pedagógicos dos teaching games for understanding e da pedagogia não-linear no ensino da educação física. Movimento.

[CR11] Clemente FM, Martins FM, Mendes RS (2014). Developing aerobic and anaerobic fitness using small-sided soccer games: methodological proposals. Strength Cond J.

[CR12] Clemente FM, Wong DP, Martins FML, Mendes RS (2014). Acute effects of the number of players and scoring method on physiological, physical, and technical performance in small-sided soccer games. Res Sports Med (Print).

[CR13] Correia V, Araújo D, Duarte R, Travassos B, Passos P, Davids K (2012). Changes in practice task constraints shape decision-making behaviours of team games players. J Sci Med Sport.

[CR14] Davids K, Button C, Araújo D, Renshaw I, Hristovski R (2006). Movement models from sports provide representative task constraints for studying adaptive behavior in human movement systems. International Society for Adaptive Behavior.

[CR15] Davids K, Araújo D, Correia V, Vilar L (2013). How small-sided and conditioned games enhance acquisition of movement and decision-making skills. Exerc Sport Sci Rev.

[CR16] Dicks M, Davids K, Button C (2008). Representative task designs for the study of perception and action in sport. Int J Sport Psychol.

[CR17] Ericsson KA (1996). The role of deliberate practice in the acquisition and maintenance of expert performance. Int J Psychol.

[CR18] García-López LM, Contreras Jordán OR, Penney D, Chandler TJL (2009). The role of transfer in games teaching: implications in the development of the sports curriculum. Eur Phys Educ Rev.

[CR19] González-Víllora S, García-López LM, Pastor-Vicedo JC, Contreras-Jordán OR (2011). Tactical awareness and decision making in youth football players 10 years: a descriptive study. Rev Psicol Depor.

[CR20] González-Víllora S, García-López LM, Pastor-Vicedo JC, Contreras-Jordán OR (2011). Tactical knowledge and decision making in young football players (10 years old). Rev Psicol Depor.

[CR21] González-Víllora S, García-López LM, Contreras-Jordán OR (2015). Evolución de la toma de decisiones y la habilidad técnica en fútbol/Decision Making and Technical Skills Evolution in Football. RIMCAFD.

[CR22] González-Víllora S, Serra-Olivares J, Pastor-Vicedo JC, Teoldo I (2015). Review of the tactical evaluation tools for youth players, assessing the tactics in team sports: football. SpringerPlus.

[CR23] Gréhaigne JF, Godbout P (1995). Tactical knowledge in team sports from a constructivist and cognitivist perspective. Quest.

[CR24] Gréhaigne JF, Bouthier D, David B (1997). Dynamic-system analysis of opponent relationship in collective actions in football. J Sports Sci.

[CR25] Gréhaigne JF, Richard JF, Griffin L (2005). Teaching and learning team sports and games.

[CR26] Griffin L, Butler J (2005). Teaching games for understanding: theory, research, and practice.

[CR27] Gutiérrez-Díaz D, González-Víllora S, García-López LM, Mitchell S (2011). Differences in decision making between experienced and inexperienced invasion games players. Percept Mot Skills.

[CR28] Gutiérrez-Díaz D, González-Víllora S, García-López LM, Mitchell S (2011). Differences in decision-making between experienced and inexperienced invasion games players. Percept Mot Skills.

[CR29] Hastie PA, Sinelnikov OA, Guarino AJ (2009). The development of skill and tactical competencies during a season of badminton. Eur J Sport Sci.

[CR30] Helsen WF, Starkes JL (1999). A multidimensional approach to skilled perception and performance in sport. Appl Cognit Psychol.

[CR31] Kelso JAS (2012). Multistability and metastability: understanding dynamic coordination in the brain. Philos Trans R Soc B Biol Sci.

[CR32] Kugler PN, Kelso JAS, Turvey MT, Kelso JAS, Clark E (1982). On the control and coordination of naturally developing systems. The development of movement control and coordination.

[CR33] Lee MCY, Chow JY, Komar J, Tan CWK, Button C (2014). Nonlinear pedagogy: an effective approach to cater for individual differences in learning a sports skill. PLoS One.

[CR34] Light R (2003). The joy of learning: emotion and learning in games through TGfU. J Phys Educ N Z.

[CR35] Mann DTY, Williams AM, Ward P, Janelle CM (2007). Perceptual-cognitive expertise in sport: a meta-analysis. J Sport Exerc Psychol.

[CR36] Memmert D (2010). Testing of tactical performance in youth elite soccer. J Sports Sci Med.

[CR37] Memmert D, Roth K (2007). The effects of non-specific and specific concepts on tactical creativity in team ball sports. J Sports Sci.

[CR38] Mitchell SA, Oslin JL, Griffin LL (2006). Teaching sport concepts and skills: a tactical games approach.

[CR39] Newell KM, Wade MG (1986). Constraints on the development of coordination. Motor development in children: aspects of coordination and control.

[CR40] Passos P, Milho J, Fonseca S, Borges J, Araújo D, Davids K (2008). Interpersonal distance regulates functional grouping tendencies of agents in team sports. J Mot Behav.

[CR41] Pinder RA, Davids KW, Renshaw I, Araújo D (2011). Representative learning design and functionality of research and practice in sport. J Sport Exerc Psychol.

[CR42] Renshaw I, Oldham ARH, Davids K, Golds T (2007). Changing ecological constraints of practice alters coordination of dynamic interceptive actions. Eur J Sport Sci.

[CR43] Renshaw I, Davids KW, Shuttleworth R, Chow JY (2009). Insights from ecological psychology and dynamical systems theory can underpin a philosophy of coaching. Int J Sport Psychol.

[CR44] Renshaw I, Chow JY, Davids K, Hammond J (2010). A constraints-led perspective to understanding skill acquisition and game play: a basis for integration of motor learning theory and physical education praxis?. Phys Educ Sport Pedagog.

[CR45] Ruiz-Pérez LM, Sánchez M, Durán J, Jiménez C (2006). Los expertos en el deporte: Su estudio y análisis desde una perspectiva psicológica. Anal Psicol.

[CR46] Serra Olivares J, González Víllora S, García López LM (2011). Game performance differences in 8–9 years football players in two 3 vs. 3 modified games. Cuad Psicol Depor.

[CR47] Serra-Olivares J, González-Víllora S, García-López LM (2015). Effects of the modification of task constraints in 3 vs. 3 small-sided soccer games. S Afr J Res Sport Phys Educ Recreat.

[CR48] Serra-Olivares J, González-Víllora S, García-López LM, Araújo D (2015). Game-based approaches’ pedagogical principles: exploring task constraints in youth soccer. J Hum Kinet.

[CR49] Starkes JL, Ericsson KA (2003). Expert performance in sports: advances in research on sport expertise.

[CR50] Stratton G, Reilly T, Williams AM, Richardson D (2004). Youth soccer: from science to performance.

[CR51] Tan CWK, Chow JY, Davids K (2012). “How does TGfU work?”: examining the relationship between learning design in TGfU and a nonlinear pedagogy. Phys Educ Sport Pedagog.

[CR52] Teoldo I, Garganta J, Greco PJ, Mesquita I (2009). Tactical principles of soccer game: concepts and application. Motriz.

[CR53] Travassos B, Araújo D, Vilar L, McGarry T (2011). Interpersonal coordination and ball dynamics in futsal (indoor football). Hum Mov Sci.

[CR54] Travassos B, Araújo D, Duarte R, McGarry T (2012). Spatiotemporal coordination behaviors in futsal (indoor football) are guided by informational game constraints. Hum Mov Sci.

[CR55] Unnithan V, White J, Georgiou A, Iga J, Drust B (2012). Talent identification in youth soccer. J Sports Sci.

[CR56] Vickers JN (2007). Perception, cognition and decision training: the quiet eye in action.

[CR57] Vilar L, Araújo D, Davids K, Travassos B (2012). Constraints on competitive performance of attacker-defender dyads in team sports. J Sports Sci.

[CR58] Vilar L, Araújo D, Travassos B, Davids K (2014). Coordination tendencies are shaped by attacker and defender interactions with the goal and the ball in futsal. Hum Mov Sci.

[CR59] Ward P, Williams AM (2003). Perceptual and cognitive skill development in soccer: the multidimensional nature of expert performance. J Sport Exerc Psychol.

[CR60] Ward P, Williams AM, Loran DFC (2000). The development of visual function in elite and sub-elite soccer players. Int J Sports Vis.

[CR61] Webb P, Pearson P (2008) An integrated approach to teaching games for understanding (TGfU). 1st Asia pacific sport in education conference. Adelaide

[CR62] Williams AM, Davids K, Williams JG (1999). Visual perception and action in sport.

